# Diagnosing metabolic syndrome in a multi-ethnic country: is an ethnic-specific cut-off point of waist circumference needed?

**DOI:** 10.1038/s41387-020-0123-8

**Published:** 2020-06-08

**Authors:** Dicky L. Tahapary, Dante S. Harbuwono, Em Yunir, Pradana Soewondo

**Affiliations:** 1 Division of Endocrinology and Metabolism, Department of Internal Medicine, Dr. Cipto Mangunkusumo National Referral Hospital, Faculty of Medicine Universitas Indonesia, Jakarta, Indonesia; 2 Metabolic, Cardiovascular and Aging Cluster, The Indonesian Medical Education and Research Institute, Faculty of Medicine Universitas Indonesia, Jakarta, Indonesia

**Keywords:** Obesity, Metabolic syndrome, Obesity, Metabolic syndrome

## Abstract

The definition of Metabolic Syndrome (MS) required an ethnic-specific cut-off point for waist circumference (WC). We aim to assess the optimal ethnic-specific WC cut-off point for MS in Indonesia, a multi-ethnic country. Three population-based studies in Indonesia were included for analysis [Flores (*n* = 1227, Floresian), Depok (*n* = 904, Sundanese), and Jakarta (*n* = 1574, Javanese)]. All subjects were 25–65 years old. The receiver operator characteristic curve analysis and Youden index method was used to determine the optimal cut-offs of WC to predict two or more risk factors of MS. In Flores, the cut-offs were 80 cm (Sensitivity and Specificity, AUC, 84% and 73%, 0.86) and 77 cm (86% and 68%, 0.85), for men and women, respectively. While in Depok, the values were 87 cm (87% and 67%, 0.85) and 79 cm (94% and 54%, 0.79), for men and women, respectively. While in Jakarta, the values were 83 cm (92% and 60%, 0.85) and 81 cm (84% and 60%, 0.77), for men and women, respectively. The optimal WC cut-off values for MS were different in those three ethnicities, and in general were lower than the currently used cut-off points for Asian population.

## Background

The prevalence of metabolic syndrome (MS), a cluster of risk factors for cardiovascular disease (CVD) and type 2 diabetes mellitus (T2DM)^1^, is increasing and is currently affecting around one quarter of the world population^2^. In Indonesia, a country with more than quarter billion population and diverse ethnicities, over the past few decades, there has been an enormous increase in the prevalence MS. Prevalence of obesity (body mass index ≥ 25 kg/m^2^) and central obesity (waist circumference (WC) ≥ 90 for men and ≥80 for women) were 23.1% and 28%, respectively^3^. In addition, the prevalence of MS in elderly increased from 21.6% in 2008 to 23.3% in 2019 (refs. ^4,5^, with the prevalence of MS in overall population was 21.6%^5^.

Several clinical definitions of MS have been proposed and widely used over past decade, including World Health Organisation (WHO)^6^, National Cholesterol Education Programme Adult Treatment Panel III (NCEP ATP III)^7^, International Diabetes Federation (IDF)^8^, and American Heart Association/National Heart, Lung, and Blood Institute (AHA/NHLBI)^9^. The main difference concerns the measure for central obesity^1^. The new definition of MS^1^ has proposed ethnic-specific cut-off values for WC, namely 90 and 80 cm for Asian men and women, respectively, which are currently used in Indonesia. However, even in Asian ethnicity, there are also differences in the cut-off points used in different Asian countries, such as China and Japan^1,10–12^. Thus, ethnic differences in Asian countries might also lead to a different cut-off values for central obesity.

As a multi-ethnic country of more than a quarter billion people and more than 600 ethnicities^13^, it will be important for Indonesia to have a country-specific or even an ethnic-specific WC cut-off points applied for the Indonesian population. Our study aims to assess the optimal WC cut-off points for the detection of MS in three different areas with different ethnicities in Indonesia. We hypothesized that each ethnic group would have each own specific WC cut-off points.

## Methods

This study used secondary data from three population-based studies in Indonesia, one from Nangapanda, Flores (*n* = 1227, mostly Floresian), one from Depok, West Java (*n* = 904, mostly Sundanese), and one from Jakarta (*n* = 1574, mostly Javanese)^14^. All subjects were between 25–65 years old and consented in this study. Incomplete data were excluded. This study was approved by the Ethical Committee of Faculty of Medicine Universitas Indonesia (1222/UN2.F1/ETIK/2018).

We used the new harmonisation criteria of MS^1^ to diagnose MS in the three areas, of which each individual need to have at least three of five MS criteria to be diagnosed as having MS. To find the cut-off value for central obesity, subjects were defined as having multiple risk factors of MS if they fulfilled two or more of the new harmonisation criteria of MS^1^ after excluding the central obesity criterion^1^: triglycerides ≥ 150 mg/dL^2^, HDL-cholesterol < 40 mg/dL in men and <50 mg/dL in women^3^, systolic blood pressure ≥ 130 mmHg and or diastolic blood pressure ≥ 85 mmHg^4^, and fasting plasma glucose ≥ 100 mg/dL. Subjects who were currently treated for dyslipidaemia, hypertension, or T2DM were deemed as having the respective risk factors, regardless of the biochemical values.

The receiver operator characteristic (ROC) curve for WC to predict the presence of two or more risk factors of the MS was plotted using plotROC package (R software). To determine the optimal cut-off point we used Youden index method (OptimalCutpoints package, R software). We also compared these results with the results from the ROC curve analysis using IBM Statistics SPSS version 23.0 of which the optimal cut-off point was determined after manually calculating the highest Youden’s index (sensitivity + specificity − 1).

## Results

Our study included a total of 3705 subjects, of which 1227 subjects, 904 subjects, and 1574 subjects were from Flores, Depok, and Jakarta, respectively (Table 1). Majority of subjects in all those areas were women.

**Table 1:** Baseline characteristics.

Variable	Flores (*n* = 1219)		Depok (*n* = 903)		Jakarta (*n* = 1574)	
	Men (*n* = 472)	Women (*n* = 755)	Men (*n* = 353)	Women (*n* = 550)	Men (*n* = 620)	Women (*n* = 954)
Age (years)	45.8 (37.8–53.4)	43.6 (34.3–51.9)	50.0 (40.0–59.0)	46.0 (38.0–53.0)	45.0 (36.0–53.0)	44.0 (36.0–52.0)
BMI (kg/m^2^)	22.1 (19.7–25.1)	23.0 (20.5–26.1)	24.5 (21.6–27.2)	25.2 (22.1–28.2)	24.2 (21.2–26.7)	25.5 (22.2–28.8)
WC (cm)	78.0 (69.8–87.0)	78.0 (70.0–87.3)	86.0 (78.0–93.0)	81.0 (74.0–88.0)	84.3 (76.2–92.0)	82.5 (75.0–90.0)
SBP (mmHg)	126.7 (116.3–140.9)	123.7 (113.7–139.3)	130.0 (120.0–150.0)	130.0 (119.4–147.5)	128.5 (117.1–144.9)	124.7 (112.5–141.6)
DBP(mmHg)	76.3 (69.8–84.3)	75.0 (69.3–84.0)	85.0 (77.5–95.0)	85.0 (75.0–92.5)	76.5 (69.0–86.0)	76.5 (68.5–85.5)
FBG (mg/dL)	93.6 (86.4–102.2)	93.6 (88.2–102.6)	89.0 (83.0–98.0)	86.0 (81.0–94.0)	88.0 (81.0–96.0)	86.0 (80.0–95.0)
TC (mg/dL)	185.4 (162.8–209.6)	193.0 (169.8–221.6)	210.0 (189–238.3)	218.0 (194.0–243.3)	199.0 (174.0–226.0)	200.0 (175.0–232.0)
HDL–C (mg/dL)	40.2 (33.3–48.7)	48.3 (40.6–58.0)	48.0 (42.0–55.0)	58.0 (51.0–66.0)	46.0 (41.0–53.0)	56.0 (48.0–64.0)
LDL–C (mg/dL)	114.3 (96.7–135.0)	119.5 (96.7–142.7)	130.0 (111.8–155.0)	135.5 (113.8–158.0)	131.0 (112.0–154.0)	130.0 (110.0–154.3)
TG (mg/dL)	131.5 (101.9–173.1)	112.5 (86.8–150.6)	131.0 (87.0–194.0)	105.0 (74.0–150.0)	128.0 (92.0–192.7)	102.0 (75.0–152.0)

All data are presented in median and its interquartile range.

*BMI* body mass index, *WC w*aist circumference, *SBP* systolic blood pressure, *DBP* diastolic blood pressure, *FBG* fasting blood glucose, *TC* total cholesterol, *HDL*–*C* high-density lipoprotein cholesterol, *LDL–C* low-density lipoprotein Cholesterol, *TG t*riglycerides.

**Fig. 1:**
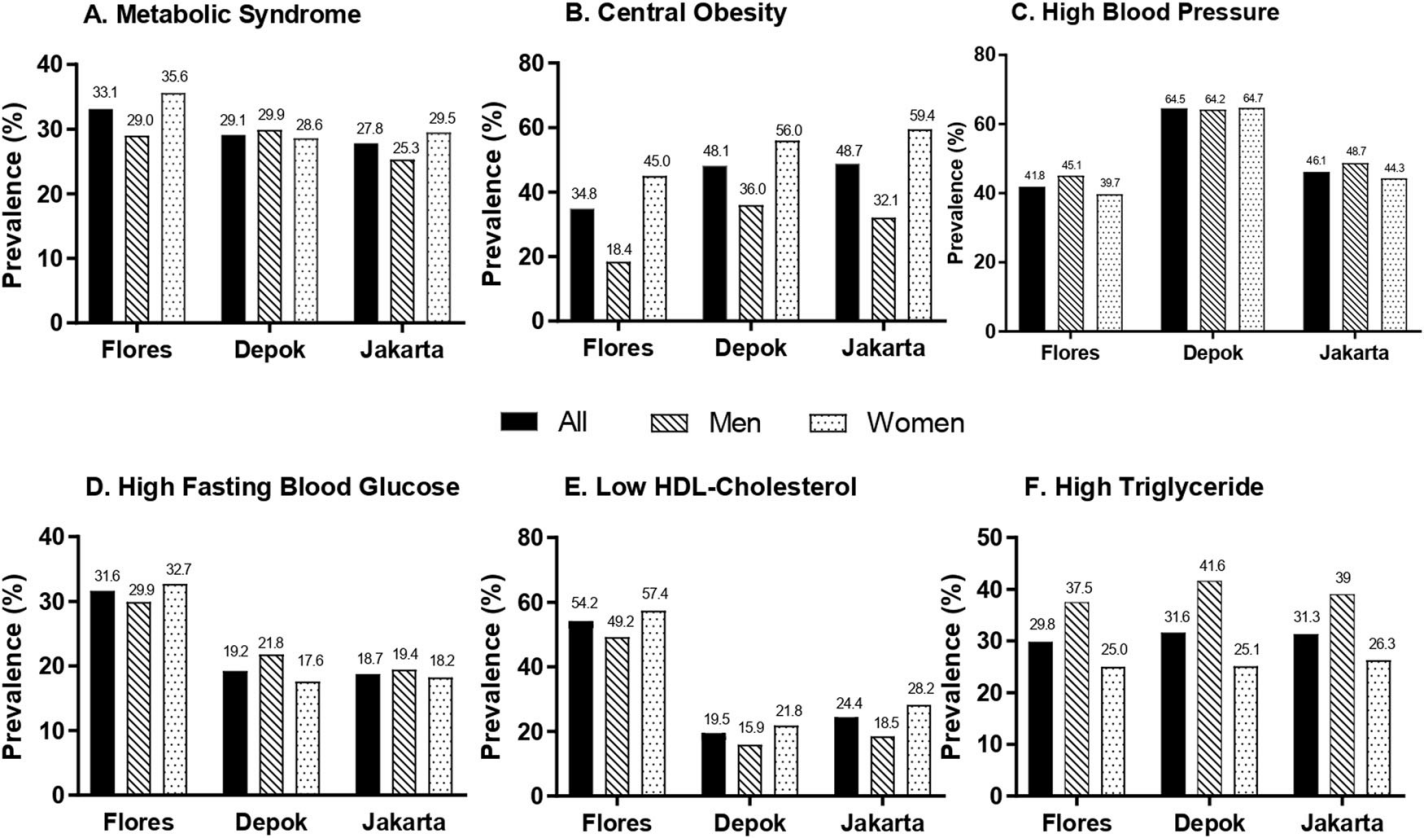
Prevalence of metabolic syndrome and its components. The prevalence of metabolic syndrome (**a**), central obesity (**b**), high blood pressure (**c**), high fasting blood glucose (**d**), low high-density lipoprotein cholesterol (**e**), and high triglyceride (**f**) in three areas (Flores, Depok, Jakarta) are presented in percentage (%).

Using the current WC cut-off points, prevalence of central obesity in Flores, Depok, and Jakarta were 34.8%, 48.1%, and 48.7%, respectively, while the prevalence of MS were 33.1%, 29.1%, and 27.8%, respectively (Fig. 1). While women had a two-times higher prevalence of central obesity in those three areas, the prevalence of MS was only slightly higher in Flores and Jakarta, and even in Depok the prevalence of MS was lower in women. The main component of MS for Flores were low HDL–C and high FBG, while for Depok and Jakarta, the main components were hypertension and central obesity.

Defining new WC cut-off for the diagnosis of MS in three different population yielded different cut-off points. In Flores, the WC cut-off was 80 cm (Sensitivity, Specificity, AUC; 84%, 73%, 0.86) and 77 cm (86%, 68%, 0.85), for men and women respectively. In Depok, the WC cut-off was 87 cm (87%, 67%, 0.85) and 79 cm (94%, 54%, 0.79), for men and women respectively. While in Jakarta, the WC cut-off point was 83 cm (92%, 60%, 0.85) and 81 cm (84%, 60%, 0.77), for men and women, respectively. Relatively similar cut-off points were observed using either the R software or SPSS (Table S1).

When we looked into the WC cut-off point for each component of MS, the range of WC cut-off point from each of the four MS criteria in Flores were 76–78 cm and 76–77 cm, for men and women, respectively (Table S2). In Depok, they were 84–87 cm and 78–81 cm, for men and women, respectively, while in Jakarta, they were 84–85 cm and 81–82 cm, for men and women, respectively (Table S2).

## Discussion

Our study observed that applying the current Asian WC cut-off would lead to a relatively similar MS prevalence in the three different ethnic populations. This despite the fact that the prevalence of central obesity varies greatly, thus potentially leads to an imprecise estimate of MS. The WC cut-off differs between the three ethnic groups in Indonesia, and the cut-off points were relatively lower than the currently used WC cut-off points for general Asian population^1,10,11^.

In Flores ethnic group, the WC cut-off value was much lower than the currently used definition of central obesity^1,11^. It is also important to note that there is not much differences between men and women. Similar findings were observed in Jakarta. In Depok, we found a relatively similar with the currently used WC cut-off point. When we look into the cut-off point for each component of MS, similar pattern was observed. Despite the differences in the cut-off points, all of the new cut-off points yield a good sensitivity to detect the presence of at least two metabolic abnormalities associated with MS, ranging from 84% to 92%. Considering that early detection of MS is of a more importance^15^, then having a better sensitivity WC cut-off might be a better approach.

Many studies support the fact that Asian has lower cut-off points than European, African, American, and Hispanic^10^. The currently used WC cut-off points for central obesity in Indonesia was derived from the population study in Japan of which a WC higher than 80 cm in women and 90 cm in men translated with a higher risk to develop T2DM and CVD in the future^16,17^. Applying this cut-off across Indonesia might then missed a quite number of people with MS. The differences in ethnicity might play a role in the differences of WC cut-off value^10,18^. Indeed, it has been reported that different ethnicity in Indonesia was associated with different body composition^19^, thus applying the same cut-off points for all ethnicities might translate to a lower or higher rate for MS.

Establishing ethnic-specific WC might be considered worthwhile; however, the practicality of their implementation should be weighed. Defining ethnicity among and within populations was proved to be challenging, especially in areas with significant representation of several ethnic groups and even sub-ethnic groups, such as in urban and suburban area. This study presumed that the main ethnic in Jakarta was Javanese, while in Depok, the main ethnic was presumed as Sundanese^14^. However, in area with ethnic homogeneity, such in rural area of Flores, the use of an ethnic-specific cut-off is likely to be more feasible.

Despite being the first study to compare WC cut-off values between three different ethnicities in Indonesia, our study has some limitations. First, the main outcome to define the cut-off point for central obesity in this study was using the presence of at least two metabolic abnormalities of MS. This approach was used considering the individual would have at least three of five MS criteria after adding the central obesity. However, it may cause bias in the diagnosis of MS. A better alternative would be to use the development of T2DM and CVD in long term follow-up as the main outcome to define the cut-off points for central obesity. Next, we did not really compare a pure ethnicity as we actually compare three populations with each area dominated by a single ethnicity^14^.

In summary, our study observed that in our three areas with different ethnicities, the WC cut-off values for MS were different, and in general were lower than the currently used cut-off points for Asians. Our finding adds to existing evidence that WC cut-off points should be adjusted for Indonesian population. Information derived from large longitudinal nation-wide studies will be needed to define a specific WC cut-off values to predict the future risk of T2DM and CVD in Indonesia.

## Supplementary information

Comparison of Waist Circumference Cut Off Points Method
