# Supplementary material for: Diagnosing metabolic syndrome in a multi-ethnic country: is an ethnic-specific cut-off point of waist circumference needed?
Source: Nutr Diabetes. 2020 Jun 8;10:19. doi: 10.1038/s41387-020-0123-8 (PMC7280283; doi:10.1038/s41387-020-0123-8)
Supplement: Supplementary file 1 — Comparison of Waist Circumference Cut Off Points Method [file 41387_2020_123_MOESM1_ESM.docx]

**Table S1. Waist Circumference Cut Off Points for > 2 Components of MS Based on Youden index and SPSS**

|  | R- Software* | | SPSS** | | J-Index (SPSS) | |
| --- | --- | --- | --- | --- | --- | --- |
|  | Men | Women | Men | Women | Men | Women |
| Flores | 80 | 77 | 79 | 77 | 0.55 | 0.54 |
| Depok | 87 | 79 | 87 | 80 | 0.53 | 0.52 |
| Jakarta | 83 | 81 | 86 | 81 | 0.53 | 0.43 |

*To determine the optimal cut off point we used Youden index method (OptimalCutpoints package, R software).

**The optimal cut off point was determined after manually calculating the Youden’s index (sensitivity + specificity -1).

**Table S2. Waist Circumference Cut Off Points for Each Component of The Metabolic Syndrome Criteria**

| Metabolic syndrome  component | Flores | | Depok | | Jakarta | |
| --- | --- | --- | --- | --- | --- | --- |
|  | Cut off (cm)  Sensitivity and specificity, AUC | | Cut off (cm)  Sensitivity and specificity, AUC | | Cut off (cm)  Sensitivity and specificity, AUC | |
|  | Men | Women | Men | Women | Men | Women |
| High TG | 77  75% and 63%, 0.74 | 77  73% and 56%, 0.70 | 85  80% and 59%, 0.75 | 81  73% and 56%, 0.66 | 84  73% and 60%, 0.71 | 81  74% and 52%, 0.68 |
| Low HDL- Cholesterol | 76  68% and 57%, 0.68 | 76  67% and 57%, 0.67 | 87  65% and 54%, 0.63 | 81  65% and 53%, 0.60 | 85  64% and 54%, 0.62 | 81  61% and 50%, 0.57 |
| High blood pressure | 76  68% and 56%, 0.65 | 77  67% and 54%, 0.64 | 84  71% and 58%, 0.69 | 78  74% and 57%, 0.71 | 84  65% and 59%, 0.64 | 81  70% and 55%, 0.66 |
| High fasting blood glucose | 78  61% and 53%, 0.60 | 77  66% and 52%, 0.63 | 86  67% and 51%, 0.65 | 81  71% and 53%, 0.63 | 84  63% and 52%, 0.62 | 82  66% and 52%, 0.63 |
| Average Cut off (cm) | 76.75 | 76.75 | 85.5 | 80.25 | 84.25 | 81.25 |
